# Linkage disequilibrium measures for fine-scale mapping of disease loci are revisited

**DOI:** 10.3389/fgene.2013.00228

**Published:** 2013-11-05

**Authors:** Carlos Zapata

**Affiliations:** Department of Genetics, University of Santiago de CompostelaSantiago de Compostela, Spain

**Keywords:** genome-wide association studies, linkage disequilibrium and genetic recombination, linkage disequilibrium between diallelic loci, new disease mutations, 2 × 2 contingency tables

Genome-wide association (GWA) studies for fine-scale mapping of disease loci are conceptually based on the occurrence and history of non-random association of alleles at different loci in populations (or linkage disequilibrium). Thus, a new disease mutation arises on a single ancestral haplotype of a given population in complete linkage disequilibrium with syntenic polymorphic markers. Owing to historical recombination events, markers close to the disease locus will tend to be in strongest disequilibrium than distant markers when the presence of evolutionary mechanisms generating non-uniform disequilibrium patterns is ignored. Therefore, the use of optimal measures to assess gradients of the strength of disequilibrium along chromosomes is crucial for fine mapping of disease loci. In practice, an ideal disequilibrium measure for fine-scale mapping should be a monotone function of the recombination fraction between the marker and the disease locus.

In an influential paper, Devlin and Risch (1995, henceforth referred to as “D&R”) compared the efficiency of five measures of linkage disequilibrium used to refine the location of disease loci: the robust formulation of the population attributable risk δ (Lewin and Bertell, 1978), Lewontin's D' (Lewontin, 1964), the correlation coefficient Δ (Hill and Robertson, 1968; also described in the literature as r), Yule's Q (Nei and Li, 1980), and Kaplan and Weir's d (Kaplan and Weir, 1992). It was concluded that the measures δ and D' outperform all other measures for fine-scale mapping. In particular, D&R established analytically that δ is the ideal measure for fine mapping because changes of δ over generations are directly related to the recombination fraction (θ) between the disease and the marker locus. In contrast, they found that D' not only depends on θ, but also on the haplotype frequencies. This supposed advantage of δ over D' for fine-scale mapping of disease loci has become a classical paradigm in the literature on disequilibrium. Nevertheless, D&R's conclusion about the relative merits of δ and D' for fine-scale mapping based on analytic results is erroneous. As is shown below, the source of the error lies in a misinterpretation of the formulas used by D&R to assess the relationship between the decay of disequilibrium by recombination and the measures δ and D'. Despite the time elapsed, this misconception remains uncorrected.

Following D&R, let us consider that there are two alleles at each of two loci: a disease allele and a normal allele segregate at the first locus, and two marker alleles segregate at the other locus. The layout and notation for population haplotype, marker allele and disease allele frequencies are given in Table 1. The measures δ and D' are defined as δ = *D*/π_+1_π_22_ and *D*' = *D*/*D*_max_, respectively; where *D* = π_11_π_22_ − π_12_π_21_ and *D*_max_ is the lesser of π_1+_π_+2_ and π_+1_π_2+_ when *D* > 0, or the lesser of π_1+_π_+1_ and π_2+_π_+2_ when *D* < 0. Under a deterministic population model, D&R assumed initial complete linkage disequilibrium between disease and marker loci; no change of disease allele and marker allele frequencies over time; the disease allele arose in a haplotype carrying the allele *A*_1_ at the marker locus, so that π_21_ = 0, π_11_ = π_+1_, π_22_ = π_2+_ and *D* > 0 at generation 0; and θ between the disease and the marker locus is constant along generations. After n generations, the decay of the initial disequilibrium (*D*_0_) can be obtained as a function of θ by means of the expression *D*_*n*_ = (1 − θ)^*n*^*D*_0_, where *D*_*n*_ is the value of disequilibrium in the nth generation (Hedrick, 2005). Expressing the measures δ and D' in terms of (1 − θ)^*n*^, D&R obtained then the following mathematical relationship:

(1) $$\begin{array}{l} {\left(1-\theta \right)}^{n}=D_{n}/D_{0}\\ \;=\left\lfloor \pi_{11}\pi_{22}-\pi_{12}\pi_{21}\right\rfloor /\pi_{+1}\pi_{22}=\delta \end{array}$$

where π_+1_π_22_ was considered to be the best estimate of *D*_0_, the initial amount of linkage disequilibrium, given that π_21_ = 0 at generation 0 and hence π_11_ = π_+1_. In contrast:

(2) $${\left(1-\theta \right)}^{n}=D^{\prime }\left[1+\left(\pi_{21}/\pi_{22}\right)\right]$$

D&R concluded, therefore, that the measure δ is a function of θ only, whereas the relationship between D' and θ depends on haplotype frequencies. Nevertheless, the formulas (1) and (2) are conceptually erroneous. Any measure of the strength of disequilibrium is computed from haplotype frequencies at a given generation. Note, however, that the numerator and the denominator in the formula (1) refer to haplotype frequencies at generations *n* and 0, respectively. As initial complete linkage disequilibrium between disease locus and marker locus decays from *D* > 0 to *D* = 0, coupling (π_11_ and π_22_) and repulsion (π_12_ and π_21_) haplotype frequencies decrease and increase, respectively, by a proportion θ each generation. Accordingly, haplotype frequencies at generations 0 and n are distinct and they should be denoted differently. Distinguishing haplotype frequencies at generations 0 and *n* by π_*ij*_(0) and π_*ij*_(*n*), respectively, where *i*, *j* = {1, 2}; the formula (1) can be then rewritten as follows:

$$\begin{array}{l} {\left(1-\theta \right)}^{n}=D_{n}/D_{0}\\ \;=\left\lfloor \pi_{11}\left(n\right)\pi_{22}\left(n\right)-\pi_{12}\left(n\right)\pi_{21}\left(n\right)\right\rfloor /\pi_{+1}\pi_{22}\left(0\right)=\delta \left[\pi_{22}\left(n\right)/\pi_{22}\left(0\right)\right] \end{array}$$

Therefore, the relationship between δ and θ depends on the frequencies of the haplotype π_22_ at generations *n* and 0. In terms of D', the formula (1) can be rewritten as:

$$\begin{array}{l} {\left(1-\theta \right)}^{n}=D_{n}/D_{0}\\ \;=D_{n}/\pi_{+1}\pi_{22}(0)\\ \;=D_{n}/\pi_{+1}\pi_{2+}\\ \;=D_{n}/D_{\text{max}}={D^{\prime }}_{n} \end{array}$$

given that π_22_(0) = π_2+_ and *D*_max_ = π_+1_π_2+_ when *D* > 0 and π_+1_π_2+_ < π_1+_π_+2_. It is shown, therefore, that D' is directly related to the recombination fraction. Likewise, the formula (2) can be rewritten as:

$${\left(1-\theta \right)}^{n}={D^{\prime }}_{n}\left\{1+\left[\pi_{21}\left(0\right)/\pi_{22}\left(0\right)\right]\right\}={D^{\prime }}_{n}$$

given that π_21_(0) = 0. D&R studied the relationship between D' and θ when π_1+_ < π_+1_. Note, however, that π_1+_ = [π_11_(0) + π_12_(0)] cannot be lower than π_+1_ = [π_11_(0) + π_21_(0)] = π_11_ (0).

**Table 1:** **Layout and notation for population haplotypes, marker alleles, and disease allele frequencies in a 2 × 2 table**.

**Marker**	**Disease allele**	**Normal allele**	
*A*_1_	π_11_	π_12_	π_1+_
*A*_2_	π_21_	π_22_	π_2+_
	π_+1_	π_+2_	1

Our reanalysis on the relationship of the recombination fraction with the measures of disequilibrium δ and D' thus, contradicts the contention by D&R that δ outperforms D' for fine-scale mapping because changes of δ over generations are directly related to the recombination fraction. In fact, we reached the opposite conclusion to that of D&R. This finding reinforces the view that D' exhibits better statistical properties as a general measure of linkage desequilibrium than other commonly used measures (Zapata, 2011). In particular, D' seems to be an optimal measure for mapping of marker association and localization of disease loci sensu D&R.
